# CAR-T Cells Shoot for New Targets: Novel Approaches to Boost Adoptive Cell Therapy for B Cell-Derived Malignancies

**DOI:** 10.3390/cells11111804

**Published:** 2022-05-31

**Authors:** Katsiaryna Marhelava, Marta Krawczyk, Malgorzata Firczuk, Klaudyna Fidyt

**Affiliations:** 1 Department of Immunology, Medical University of Warsaw, 02-097 Warsaw, Poland; katsiaryna.marhelava@wum.edu.pl (K.M.); marta.krawczyk@wum.edu.pl (M.K.); mfirczuk@wum.edu.pl (M.F.); 2 Laboratory of Immunology, Mossakowski Medical Research Institute, Polish Academy of Sciences, 02-106 Warsaw, Poland; 3 Doctoral School of Translational Medicine, Mossakowski Medical Research Institute, Polish Academy of Sciences, 02-106 Warsaw, Poland

**Keywords:** CAR-T cells, adoptive immunotherapy, CD19 antigen, B-ALL, B cell lymphoma, resistance mechanism

## Abstract

Chimeric antigen receptor (CAR)-T cell therapy is undeniably a promising tool in combating various types of hematological malignancies. However, it is not yet optimal and a significant number of patients experience a lack of response or relapse after the treatment. Therapy improvement requires careful analysis of the occurring problems and a deeper understanding of the reasons that stand behind them. In this review, we summarize the recent knowledge about CAR-T products’ clinical performance and discuss diversified approaches taken to improve the major shortcomings of this therapy. Especially, we prioritize the challenges faced by CD19 CAR-T cell-based treatment of B cell-derived malignancies and revise the latest insights about mechanisms mediating therapy resistance. Since the loss of CD19 is one of the major obstacles to the success of CAR-T cell therapy, we present antigens that could be alternatively used for the treatment of various types of B cell-derived cancers.

## 1. Introduction

In the last decade, chimeric antigen receptor (CAR)-modified T cells have emerged as a breakthrough treatment for patients with relapsed/refractory (r/r) B cell-derived malignancies. Until now, B cell acute lymphoblastic leukemia (B-ALL) and B cell lymphoma patients have benefited most from this clinically-approved cellular therapy. The survival prognosis for r/r B-ALL and diffuse large B cell lymphoma (DLBCL) patients who receive conventional treatment is still not satisfactory. Before the approval of CD19 CAR-T cells, the 3-year survival rate after the first relapse for adult B-ALL was 11% [1], whereas for pediatric patients the event-free survival did not exceed 50% [2,3]. For relapsed DLBCL patients, 4-year progression-free survival varied between 20 and 40% [4]. Similar to r/r B cell cancers, plasma cell-derived malignancy—multiple myeloma (MM) is difficult to manage clinically, especially due to an increasing resistance to the available treatment regimens [5,6]. Therefore, very recently, CAR-T therapy targeting B cell maturation antigen (BCMA) was introduced for the treatment of r/r MM patients [7].

Unfortunately, despite the undeniable success of CAR-T cell therapy in hemato-oncology, there are still patients who progress after this therapy or develop resistance mechanisms that avert the eradication of malignant cells. Taking into consideration major CAR-T pitfalls, herein, we describe novel strategies aimed at boosting the antitumor effects of this highly personalized treatment. In particular, we present improvements in the area of CAR-T cells persistence and the management of effector cells exhaustion. Furthermore, we highlight the mechanisms exploited by malignant B cells to escape the CD19 CAR-T treatment. Indeed, we characterize various factors contributing to the development of resistance, both related and unrelated to the CD19 antigen loss. Lastly, we describe surface molecules that could be used as new promising targets for CAR-T adoptive therapy in B cell and plasma cell-derived blood cancers.

## 2. Clinically Available CAR-T Therapies

Currently, there are four different CD19-targeting, and one BCMA-targeting CAR-T products, approved by American and European Drug Agencies (Food and Drugs Administration and European Medicines Agency, respectively). All these products are used for the treatment of malignancies derived from abnormal B cells at different stages of their differentiation.

The general structure of a CAR construct includes four parts: (1) an extracellular antigen-binding domain, (2) a hinge, (3) a transmembrane helix, and (4) an intracellular signaling domain. Accordingly, decades of research in the area of CAR design have led to the development of five CAR-T cell generations, each of them differing in an intracellular signaling milieu composition. In fact, the first CAR-T generation contains only a single immunoreceptor tyrosine-based activation motif (ITAM) domain derived from a CD3ζ chain of the T cell receptor (TCR). Further approaches have involved adding one (second generation) or two (third generation) co-stimulatory domains (mostly CD28 and/or 4-1BB) that enhance CAR-T cells activation and persistence [8,9,10]; however, in order to additionally boost the CAR-T cells activation, proliferation, and killing potential, fourth and fifth CAR-T generations were designed [11]. Such CAR-T cells are armored with additional intracellular domains responsible for specific cytokines production (e.g., IL-12, IL-2). These CAR-T cells are currently being evaluated in the research phase.

CAR-T cells products used for therapy of hematological patients represent the second CAR-T cell generation, with either a CD28 or 4-1BB (CD137) co-stimulatory domain. Since 2017, four CAR-T cell products aimed to target CD19 were approved based on pivotal phase 1/2 clinical trials, all of which are still active. Initial clinical responses for each trial and appropriate follow-up studies results (with observations lasting longer than 2 years) are summarized in Table 1.

Out of these four products only tisagenlecleucel (Kymriah) is registered for children and young adults (up to 25 years old) with B-ALL, who are refractory to previous treatment regimens, or with more than two relapses. Considering that it was the first CAR-T cell therapy approved, there is the longest follow-up available for this product (median 4.8 years), showing complete response (CR) in more than 60% of the patients and overall survival (OS) equal to 10.5 months [13]. On the other hand, the access to CD19-targeted CAR-T therapy for adult r/r B-ALL patients was not granted until recently, when in October 2021 the FDA approved brexucabtagene autoleucel (Tecartus) for such indication. The registration was based on the multicenter ZUMA-3 trial [20,25]. The efficacy estimation group in the phase 2 ZUMA-3 trial included 55 adult previously treated patients, of which 27% represented a high-risk group, bearing BCR-ABL1 translocation [20]. CR was achieved in 56% of the patients, and more importantly, CR or complete remission with incomplete hematological recovery (CRi) was reached in 60% of the patients previously submitted to blinatumomab, another CD19-targeted immunotherapy. The side effects post CAR-T infusions were generally manageable. Taking into consideration the combined results of phases 1 and 2, the median duration of remission in this trial was estimated by the investigators at 13.4 months; however, a longer follow-up is needed to draw stronger conclusions regarding safety profiles and the durability of responses.

In addition to adult B-ALL, brexucabtagene autoleucel is also approved for the treatment of mantle cell lymphoma (MCL). The results gathered during ZUMA-2 trial showed encouraging responses in heavily pre-treated r/r MCL patients, with CR achieved in 67% after single CAR-T administration [19]. Common adverse events following CAR-T therapy occurred in the majority of treated patients, however, they were mostly manageable. A one-year follow-up of the ZUMA-2 trial presented at the 2020 ASH Annual Meeting showed that with a median of 17.5 month-long observations, the CR was maintained in 67% of the patients [26].

For the treatment of B cell lymphomas, several CAR-T products are available. Based on JULIET [14] and ZUMA-1 [16] trials, tisagenlecleucel (Kymriah) and axicabtagene ciloleucel (Yescarta), respectively, were first to be approved for the treatment of various types of B cell lymphoma, including adult r/r DLBCL, DLBCL arising from follicular lymphoma (FL), or primary mediastinal large B cell lymphoma (PMBCL), for the patients who failed after two or more lines of systemic therapy (see Table 1 for more details). Importantly, long-term follow-up of patients’ status within these trials (median follow-up exceeding 3 years) provided data showing a superior efficacy of Yescarta, with median OS reaching 25.8 months [17] as compared to 11.1 months for Kymriah [15]. This discrepancy, however, could arise from the fact that most of the patients in the JULIET study received a bridging therapy (e.g., rituximab, nucleoside analogues, corticosteroids), whereas in the ZUMA-1 trial bridging chemotherapy was not allowed, with the exception of corticosteroids administration. Thus, one can speculate that the JULIET trial included more patients with aggressive lymphomas; however, the direct impact of bridging therapies on CAR-T responses should not be excluded.

The year 2021 was extremely rich in FDA/EMA approvals for CAR-T therapies. In addition to the aforementioned Tecartus approval for r/r adult B-ALL, the additional registrations included axicabtagene ciloleucel (Yescarta) for r/r adult FL [18], and lisocabtagene maraleucel (Breyanzi) for adult r/r DLBCL, r/r PMBCL, grade 3B FL [21]. Moreover, in 2021, we witnessed the first approval of a BCMA-directed CAR-T therapy designated for adult r/r MM patients who had failed at least four previous lines of treatment [24]. Indeed, the efficacy of idecabtagene vicleucel (Abecma) was evaluated in the pivotal KarMMa clinical trial [23]. Stringent CR (sCR) was detected in only 28%, however, the CAR-T cells were administered to patients with a significant number of prior lines of treatment (median of 6 lines) [24]. Importantly, 65% of the patients who achieved sCR had a durable response for at least 12 months. All in all, CAR-T cells targeting BCMA seem to be an attractive treatment option for heavily pre-treated, relapsed MM patients. Certainly, a longer follow-up is still required to estimate the safety profiles and survival rates in a long run.

## 3. Managing Major Pitfalls of CAR-T Cell Therapy

Despite the phenomenal success of CD19 CAR-T therapy, it is not without limitations and side effects. The challenges faced by CAR-T cell therapy are related to both effector and target cells and include: (1) proliferation, persistence, and exhaustion of CAR-T cells, (2) side effects, (3) immunosuppressive tumor microenvironment, and (4) tumor intrinsic factors that account for the development of resistance.

The obstacles related to effector CAR-T cells are elegantly described by others [27,28,29,30,31]. Therefore, below we only shortly present currently investigated and implemented solutions to modulate insufficient CAR-T cells persistence in vivo, and exhaustion of the effector cells. Considering, however, that more and more data is now gathered on tumor related features contributing to immunotherapy resistance, in this review we will mostly focus on limitations and solutions concerning target cells. In particular, we portray the mechanisms responsible for resistance to CD19 CAR-T therapy, both CD19-dependent and independent (Section 4), and new antigens explored in hemato-oncology (Section 5).

### 3.1. Improvement of CAR-T Cells Persistence

The sufficient number of viable and functional CAR-T cells is one of the major contributors to the therapy’s success [16,32]. The poor persistence even after the achievement of complete remission is considered to be the main reason for the development of CD19-positive relapses [33,34]. One of the features affecting CAR-T cell persistence and proneness to exhaustion is the co-stimulatory domain incorporated in the CAR construct. CD28 and 4-1BB are the most frequently exploited co-stimulatory domains in pre-clinically tested and in clinically available CAR products (Table 1). Several studies have demonstrated a higher proliferative capacity and longer in vivo persistence of CAR-T cells bearing a 4-1BB domain as compared to CD28 [35,36]. This effect was at least partly attributed to the 4-1BB-mediated activation of non-canonical nuclear factor κB (ncNF-κB) signaling in CAR-T cells [37]; however, as CARs with a CD28 domain exhibit more rapid tumor elimination [38], attempts to improve their in vivo performance have led to a generation of mutant CD28 endodomain [39], or third generation CARs containing both CD28 and 4-1BB sequences [40]. The latter were demonstrated to retain the beneficial aspects of both domains, showing enhanced antitumor activity and concomitantly increased in vivo persistence.

In the clinical settings, in order to improve CAR-T cells persistence and thus the overall therapy efficacy, lymphodepleting chemotherapy is performed prior to CAR-T cells infusion. The pre-conditioning is most often based on fludarabine and/or cyclophosphamide administration. The reason standing behind this approach was demonstrated in several studies showing higher response rates in patients with r/r B-ALL [41,42], and non-Hodgkin lymphomas, including r/r DLBCL [43,44,45], who received lymphodepleting chemotherapy regimens prior to CD19 CAR-T cells. Primarily, lymphodepletion reduces the numbers of endogenous lymphocytes, which normally absorb the cytokines that stimulate T cells proliferation and thus limit their availability for infused CAR-T cells [44,46]. In addition, it partially eliminates T regulatory cells (Tregs) which exhibit immunosuppressive activity towards tumor-specific cytotoxic T cells [47]. The beneficial effect of lymphodepleting regimens was demonstrated for both CD8-positive and CD4-positive CAR-T cell populations [48].

However, the intensity of existing pre-conditioning therapy using fludarabine and cyclophosphamide was shown to be correlated with higher toxicity of the CAR-T cell-based therapy, thus it may exclude the application of this approach for numerous patients [49]. The approach of targeted lymphodepletion with a CD45-directed antibody radioconjugate, which was recently shown to eliminate several subsets of leukocytes while preserving progenitor hematopoietic cells in a murine model, could potentially be proposed as a safer alternative [50]. However, regardless of the lymphodepleting protocol used, the inevitable side effects of these regimens are lymphopenia and prolonged T cells dysfunction, which may lead not only to overall higher sensitivity to infections and autoimmunity development, but also to tumor relapse [51].

Some already existing approaches undermine the need for lymphodepletion. The most prominent is based on the construction of CAR-T cells that besides having the ability to recognize specific targets can also produce selected proteins, in particular cytokines [52]. These 4th generation CAR-T cells are called TRUCKs (T cells redirected for antigen-unrestricted cytokine-initiated killing). Cytokines are crucial for CAR-T cells’ survival, proliferation and persistence, both during in vitro culture and following the administration into the patient [53]. The additional modification of CD19 CAR-T cells to secrete IL-12 was shown to improve their in vivo cytotoxicity towards murine cells expressing human CD19 and alleviate their suppression by Tregs—the main two aims of pre-conditioning with chemotherapy [54]. Similar observations were made by Kueberuwa et al., who demonstrated that CD19 CAR-T cells expressing IL-12 effectively eliminated lymphoma cells in fully lymphorepleted mice [55]. Besides the killing of CD19-positive target cells directly, the TRUCKs also contributed to the induction of an anticancer response elicited by the host immune cells, primarily CD8-positive T cells.

Except for IL-12, other cytokines were also shown to improve the persistence and effector functions of transferred cytotoxic T cells. In particular, IL-2, IL-7, and IL-15 supplementation during the culture of CAR-T cells has beneficial effects [56,57]. Interestingly, CAR-T cells expanded with IL-15 exhibited a higher anticancer efficacy as compared to IL-2-supplemented CD19 CAR-T cells against a lymphoma cell line in vivo [58]. In addition, IL-15-expressing CAR-T cells appear to be less terminally differentiated and have an increased ability to expand in vitro, but also tend to be more toxic in vivo due to the simultaneously higher production of TNF-α and IL-2, as compared to CAR-T cells without IL-15 [59]. On the other hand, constitutive cytokine expression poses a risk for the uncontrolled growth of administered CAR-T cells which may lead to overt toxicity. Several research groups have demonstrated that novel CAR constructs may be engineered to induce cytokine signaling only after antigen stimulation (5th CAR-T generation) and preserve their superior antitumor effects while having minimal toxicity [60,61,62]. The additional incorporation of an inducible caspase-9-based suicide gene, which mediates the selective depletion of CAR-T cells in case of toxicity, may increase the safety while keeping all the advantages of the therapy based on using anti-CD19 TRUCKs [63,64,65].

### 3.2. Modulation of CAR-T Cells Exhaustion

The effectiveness of CAR-T cells is also impeded by their exhaustion, which is often caused by the enhanced expression of immune checkpoint molecules on cancer cells and CAR-T cells. In particular, PD-1/PD-L1 interaction activates downstream signaling pathways and inhibits T cell activation, which leads to tumor immune escape [66]. PD-L1 was reported to be overexpressed in classical Hodgkin lymphoma [67,68] and DLBCL [69]. Moreover, PD-1 expression was observed to be increased on T cells isolated from B-ALL patients’ bone marrow aspirates [70]. Similarly, CD19 CAR-T cells were also reported to have increased PD-1 expression following the infusion to the patients [71]. The solution for this issue can be achieved by combining a CAR-based therapy with anti-PD-1 antibodies [72,73], or their administration just after CAR-T failure [74]. The more advanced and safe approaches include the delivery of CAR-T cells secreting anti-PD-1 scFv [75], or the transformation of PD-1 in CAR-T cells into a co-stimulatory molecule using the switch-receptor technology [76].

Recent reports have also shown that CAR-T cells exhaustion occurs due to epigenetic reprogramming [77,78,79]. In a clinically-relevant setting, Zebley et al. observed the changes in DNA methylation patterns in CD8-positive CD19 CAR-T cells that were previously administered to B-ALL patients [79]. Importantly, a deletion of a gene coding DNA methyltransferase 3 alpha (*DNMT3A*) in T cells bearing CAR constructs augmented a long-term antitumor response and prevented them from developing an exhausted phenotype [77]. In addition, several other modifications show promise to limit CAR-T cells exhaustion. For instance, CAR-T cells engineered to overexpress a transcription factor c-Jun had an improved ability to resist exhaustion and maintain functionality despite prolonged activation [80].

There are also other factors contributing to the loss of CAR-T cells effector abilities, including those related to CAR tonic signaling [81]. One of the consequences of CAR tonic signaling is terminal differentiation to effector cells. The available literature indicates the involvement of sustained phosphoinositide 3-kinase (PI3K)/Akt pathway activation in this process [82,83]. Therefore, pharmacological approaches aimed at PI3K/Akt signaling inhibition have been implemented during the manufacturing phase to maintain CAR-T cells stemness, including the addition of tyrosine kinase inhibitors, e.g., idelalisib [84], ibrutinib [85], or duvelisib [86]. In addition, the modifications of CAR constructs may be performed to address the issue of CAR tonic signaling and involve several aspects, e.g., the substitution of co-stimulatory domains, alterations in the spacer and hinge domain, or the improvement of scFv stability [81]. An alternative approach is to reduce CAR surface expression and selectively switch-off CAR signaling. This could be achieved by incorporating the ligand-induced degradation/destabilizing domain into the C-terminus of the CAR construct [87,88]. In addition, Weber et al. modulated CAR-T cells exhaustion by the addition of dasatinib, a tyrosine kinase inhibitor that reversed the tonic signaling and led to downregulation of the immune checkpoint markers [88]. Importantly, dasatinib administration inhibits the secretion of cytokines by CAR-T cells and impairs their in vivo antitumor activity [89]. This effect, however, is reversible after the drug discontinuation, which implies that short-term dasatinib treatment could be used as a switch to control CAR-T cells’ performance.

## 4. Mechanisms of Resistance to the CD19 CAR-T Therapy in B Cell Malignancies

**Figure 1:**
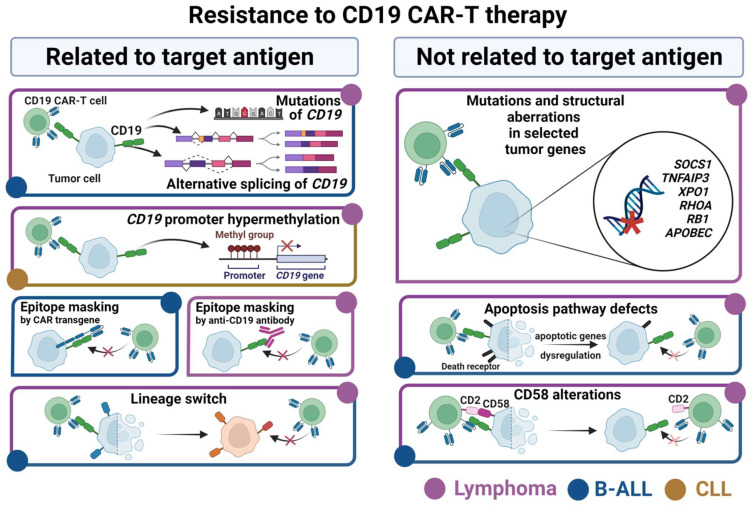
B cell-derived cancers develop various mechanisms of resistance to CD19 CAR-T therapy. Left panel: the mechanisms related to downregulation of target antigen include genetic and epigenetic aberrations within *CD19* gene or alternative splicing of *CD19* transcripts. Another possibility of resistance development is CD19 epitope masking by either a CD19-specific CAR construct, or therapeutic anti-CD19 antibody. Target antigen loss can also occur as a consequence of phenotypic changes due to the lineage switch of malignant B cell precursors. Right panel: target antigen-independent mechanisms of resistance cover structural aberrations and mutations in selected genes of malignant lymphoma cells. Additionally, disruption of the apoptosis pathway or CD58-CD2 co-stimulation could be involved in this process.

As already mentioned in Section 3, not only insufficient effector cells are to blame for CAR-T therapy failures. Intrinsic features of malignant B cells also significantly contribute to the development of resistance underlying a relapse in patients with B-ALL and high-grade lymphomas. Indeed, the relapse in B-ALL patients following CD19 CAR-T treatment can affect even half of them, but the cumulative incidence of relapse according to the meta-analysis of available clinical trials’ reports is estimated at 36% [90]. The most commonly reported causes of tumor resistance to CD19 CAR-T treatment and subsequent relapse concern various tumor cells aberrations, among which the loss of CD19 antigen is the most frequent [91]. The loss of CD19 is reported even in up to 25% of B-ALL cases [12,92,93,94,95]. The relapses after CD19 CAR-T therapy occur also in lymphomas, where about 30% of the patients demonstrate a loss of CD19 expression [16,96,97]. Moreover, in some patients the CD19 downregulation is accompanied by decreased expression of other surface antigens, such as CD20 and CD22 in B-ALL [98] and similarly CD20, CD22, and CD79a in lymphomas [99,100]. Below, we describe the currently known tumor-intrinsic mechanisms of CD19 CAR-T therapy resistance, highlighting the similarities and differences between various B cell malignancies, particularly B-ALL and B cell lymphomas (Figure 1).

### 4.1. Resistance Related to CD19 Antigen

One of the causes of the CD19 antigen loss is a mutation in the *CD19* gene and alternative splicing of the *CD19* transcript. The missense and frameshift mutations in exon 2 of the *CD19* gene, as well as alternative splice variants lacking exon 2 or exons 5–6, were detected in samples from relapsed B-ALL patients after CD19 CAR-T therapy [101]. Sotillo et al. also highlighted that the deletion of exon 2 promotes arising of the N-terminal truncated CD19 isoform, which prevents killing by CD19 CAR-T cells, due to the loss of the epitope recognized by a particular anti-CD19 scFv used in this study [101]. Moreover, *CD19* exon 2 retention was linked with a function of serine and arginine-rich splicing factor 3 (SRSF3). The reduced expression of SRSF3 and consequent N-terminally truncated CD19 was observed in samples from B-ALL patients who relapsed after CD19 CAR-T [101]. Similar results were obtained by Orlando et al. [102], where *CD19* mutations were linked to the antigen loss. The analysis of primary material from 17 B-ALL pediatric and young-adult patients with relapses after CD19 CAR-T administration revealed that 12 of them were CD19-negative. All of these 12 patients had mutations in the *CD19* gene that affected exons 2–5 and led to the expression of a truncated protein devoid of the transmembrane domain; however, some of the observed mutations could be subclonal, which is reflected by the ratio between the CD19 negative cells percentage and the rate of mutations observed in the patients’ sample [102]. Later studies showed that *CD19* splice variants lacking exon 2 were also detected in B-ALL pediatric patients at diagnosis and could be the cause of initial leukemia resistance to CD19-targeting CAR-T treatment [103]. Similar observations were made for B cell lymphoma, where CD19 isoforms with exon 2 and exons 5–6 aberrations (that could affect FMC63 epitope) were detected in patients’ samples and were found to be more common after relapse. On the other hand, the full-length isoform of CD19 was co-expressed with spliced variants in relapsed samples, suggesting that the loss of the FMC63 epitope is not the only cause of relapse [100].

Another genetic-related factor that could account for the CD19 loss is the retention of intron 2 in *CD19* mRNA. The presence of intron 2 in mature mRNA molecules can induce premature termination and decay of the *CD19* transcript or the appearance of a truncated protein. The phenomenon of intron 2 retention was revealed using lymphoma and B-ALL cell lines models and confirmed in matched diagnosis-relapse B-ALL patients’ samples [104].

Recent studies also showed that the antigen loss following CD19 CAR-T therapy can be a result of hypermethylation of the *CD19* promoter in tumor cells [105]. It was demonstrated in an in vivo chronic lymphocytic leukemia (CLL) relapse model after CD19 CAR-T treatment, as well as in vitro experiments using primary CLL cells and CLL and Burkitt lymphoma cell lines co-cultured with CD19 CAR-T cells. The correlation between *CD19* promoter hypermethylation and decreased CD19 expression was found to be reversible by demethylating drugs, highlighting the reversible character of this epigenetic modification.

In addition to CD19 loss on the surface of malignant cells, CAR-T therapy failure may be caused by CD19 epitope masking. This mechanism was identified by Ruella et al. in a B-ALL patient with relapse that was discovered almost 9 months after CD19 CAR-T treatment [106]. Unexpectedly, the researchers observed CAR-expressing leukemic blasts in the patient’s bone marrow. It turned out to be a result of unintentional CAR transgene insertion into a single neoplastic B cell during the CD19 CAR-T cells manufacturing process. As a consequence, CAR expression on the cell surface of leukemic B cells masked the CD19 epitope in cis and hampered the binding of the antigen by CAR-T cells. This phenomenon was further confirmed using a CD19 CAR-modified Nalm-6 cell line and in a xenograft model in vivo. These experiments proved that leukemic B cells expressing CAR are resistant to CD19 CAR-T cell-mediated killing [106]. This discovery contributed to modifications of the CAR-T manufacturing protocols and the development of new strategies providing maximal T cell selection [107,108]. Importantly, the CD19 epitope masking may also be due to prior CD19-targeted therapy. Indeed, Fitzgerald et al. observed that tafasitimab, an anti-CD19 antibody approved for r/r DLBCL treatment, masked the CD19 epitope and caused a delay in CD19 CAR-T therapy responses [109].

Yet another way of resistance to CD19 CAR-T cell therapy is lineage switch, which results not only in CD19 loss but also in many other phenotypic changes. It was demonstrated that some B-ALL patients after CD19 CAR-T treatment relapsed with a myeloid type of leukemia expressing CD34, CD33, or CD64 markers [110]. Moreover, lineage reprogramming in these cases was found to be correlated with the aberration of genes encoding Pax5, EBF1, or FLT3. The phenomenon of lineage switch is one of the causes of resistance to CD19 CAR-T therapy, mainly in mixed lineage leukemia (MLL)-rearranged B-ALL [111,112,113], however, one case was also detected in a Philadelphia chromosome-positive B-ALL patient [114]. Recent studies showed that a similar situation can also occur in lymphomas. Zhang et al. described the case of MCL that transdifferentiated into sarcoma [115]. It was triggered by epigenetic reprogramming, including the silencing of mature B cell characteristic genes by promoter DNA methylation. The broad analysis of samples from patients with lymphomas (mainly DLBCL) before and after CD19 CAR-T therapy displayed a tremendous phenotypic shift in tumor cells after CAR-T infusion. These genomic changes included downregulated expression of B cell markers (CD19, CD20, CD22, CD79a), an increased methylation profile, and mutations affecting immune suppressive mechanisms, such as the PI3K pathway [116].

### 4.2. Resistance Not Related to CD19 Antigen

Currently, it is known that not only CD19 aberrations can influence the response to CD19 CAR-T immunotherapy. The circulating tumor DNA profiling of samples from r/r DLBCL patients who underwent CD19 CAR-T treatment determined the association of *SOCS1*, *TNFAIP3*, and *XPO1* mutations with a poor prognosis after that therapy [117]. Additionally, the whole-genome sequencing analysis of CD19 CAR-T-treated large B cell lymphoma patients indicated some tumor intrinsic aberrations, such as *RHOA* or *RB1* deletions, APOBEC mutational activity, and chromothripsis events as characteristics for relapsed patients [118,119]. The aforementioned defects were accompanied by preserved CD19 expression.

An inherent resistance of neoplastic B cells for CD19 CAR-T therapy has been also correlated with aberrant expression of genes from the death receptor apoptosis pathway such as *BID*, *FADD*, *CASP8*, *TNFRSF10B*, *Fas*, and *TRAIL* [91,120]. The crucial role of death receptor signaling in susceptibility to CD19 CAR-T cytotoxicity was demonstrated in a Nalm-6 B-ALL cell line in vitro model and CRISPR-based genome-wide loss-of-function screen [121,122]. It was observed that Nalm-6 cells with a deletion of apoptotic genes, such as *BID* or *FADD*, diminished the CAR-T cells’ expansion and their cytotoxic abilities. Moreover, analysis of primary samples from B-ALL pediatric patients after CD19 CAR-T therapy indicated a lower expression of death receptor signaling genes in a group of patients who did not respond to the treatment as compared to those who achieved complete remissions [122]. Using the same approach based on the CRISPR-based screen, the significant role of Fas-FasL in antigen-specific T cell killing was confirmed in B cell lymphoma cell lines [123].

In addition to the aforementioned resistance mechanisms, the lack of CD58 on the surface of malignant B cells, a co-stimulatory ligand for CD2 on T cells, was also demonstrated as one of the causes of poor prognosis and resistance to CD19 CAR-T therapy. Changes in CD58, including downregulation, mutation, or loss, were observed in up to 67% of DLBCL patients [124]. CD58 aberrations were found to be correlated with a faster progression of lymphoma disease and decreased durable, complete responses to CD19 CAR-T treatment. This observation was also confirmed in in vitro and in vivo models using a Nalm-6 CD58 KO B-ALL cell line. These pre-clinical studies showed that CD19 CAR-T cells had a reduced cytokine production and killing potential after contact with CD58 KO tumor cells. Similarly, mice with B-ALL tumors lacking CD58 expression only partially responded to CD19 CAR-T treatment [125].

## 5. New CAR-T Targets Explored in Hematological Malignancies

Considering that around half of the leukemia relapses that occur following CD19 CAR-T administration are manifested by the appearance of CD19-negative cells [12,93], the development of new approaches is crucial to managing such cases. One of the strategies to eliminate the risk of the disease recurrence is to perform allogeneic hematopoietic stem cell transplantation (allo-HSCT) [126]; however, many of the patients, who are usually heavily pre-treated, are not eligible for allo-HSCT due to its toxicity and potential side effects [127]. Hence, another strategy to avoid CD19-negative relapses is focused on targeting alternative molecules expressed on the surface of malignant cells. Searching for novel antigens is still ongoing, and several targets are already being evaluated in pre-clinical and/or clinical studies [128]. Herein, we present promising targets that are currently investigated for the treatment of B cell and plasma cell-derived malignancies.

These novel (other than CD19) targets are summarized in Table 2. For each targeted molecule, we highlight its physiological functions, the expression on normal cells, and the targeted disease. Further in this section, we also describe several antigens in more detail. Primarily, we elaborate on less-explored targets that are not widely discussed in CAR-T-oriented literature reviews, such as CD32B, CD70, and CD72. We also characterized promising, clinically explored targets that are tested in blood cancers derived from B cells at various stages of development and maturation, including BAFF-R (B-ALL), CD37 (CLL, B cell lymphoma), and GPRC5D (MM).

### 5.1. Novel Antigens under Investigation in Pre-Clinical Studies

**CD32B** (FcγRIIB) is the low-affinity inhibitory receptor for IgG, which contains an intracellular immune inhibitory motif (ITIM). CD32 is expressed in three isoforms: CD32A, B, and C. The isoform B occurs in normal and malignant B cells and some subpopulations of dendritic cells and granulocytes [138]. The expression of CD32B was also detected in non-hematopoietic tissues such as liver endothelial cells [140] and airway smooth muscle cells [139]. CD32B is the main FcγR present on B cells. Following co-ligation with activating receptors such as B cell receptor (BCR), CD32B recruits SH-2 containing inositol 5′ phosphatase 1 (SHIP-1), which attenuates activating signals and contributes to the regulation of B cell activation and homeostasis [138,180,181]. High and homogenous expression of CD32B was also demonstrated in B cell-related disorders [141,182]. Moreover, it was shown that the expression of CD32B negatively affects immunotherapy of CD20-expressing lymphomas with therapeutic monoclonal antibody (mAb) rituximab [183,184]. The mechanism results from the binding and internalization of rituximab by CD32B. Antagonistic mAbs specifically targeting the CD32B isoform improved rituximab efficacy in pre-clinical lymphoma models and were further tested in combination with rituximab in lymphoma patients [185]. Recently, CAR-T cells containing scFv derived from mAbs specifically recognizing the CD32B isoform were generated and presented to efficiently kill CD32B-expressing cell lines and primary CLL cells in vitro and in vivo [141]. However, considering the expression of CD32B in normal cells, CAR constructs with improved safety such as dual CARs with an “AND” logic gate should be developed due to the potential on-target off-tumor toxicity.

**CD70**, a type II transmembrane glycoprotein from the tumor necrosis factor (TNF) family, is a ligand for the CD27 receptor present on T cells [186,187]. The CD70/CD27 interaction was shown to serve as a co-stimulatory signal for T cell activation and to play a role in T cell-dependent B cell differentiation into plasma cells [188,189]. In healthy individuals, the expression of CD70 was found to be restricted to subsets of activated T cells, B cells, NK cells, mature DCs, and epithelial cells of the thymic medulla [142,143]. In contrast to normal cells, various types of malignant lymphoid cells exhibit aberrant CD70 expression. Increased CD70 levels were observed on various malignant B cells, for instance, isolated from patients with CLL, DLBCL, MCL, and MM [190,191]. Importantly, CD70 overexpression in MCL patients was recently reported to be associated with higher proliferation, a more aggressive clinical course of the disease, and with an increased number of Tregs infiltration in the tumor [192]. The possible role of CD70 in the generation of the immune-suppressive tumor microenvironment was also demonstrated by Yang et al. [193]. In this study, CD70-positive biopsy-derived primary lymphoma B cells more potently enhanced Foxp3 levels in CD4-positive CD25−negative T cells subpopulation than their CD70-negative counterparts.

Anti-CD70 antibodies inhibited tumor growth and prolonged mice survival in models of CD70-positive B cell malignancies [191,194], providing the rationale for targeting CD70 with CAR-T cells. In 2011, Shaffer et al. demonstrated that CD70-specific T cells caused in vivo lymphoma regression [144]. Importantly, the study also evaluated the toxicity of CD70 CAR-T cells towards activated T and B cells, which physiologically express CD70 upon activation. While activated T cells were not killed in cytotoxicity assays, B cells stimulated with the CD40 ligand were observed to be eliminated by CD70 CAR-T. However, the issue of potential B cell aplasia in vivo remains uncertain as CD70 expression on normal lymphoid cells occurs only transiently. Recently, Deng et al. also showed the effectiveness of CD70 CAR-T cells against CD19 KO Raji cells in vitro and in vivo [145]. The study shows the potential application of this approach in the treatment of CD19-negative B cell malignancies, thus addressing the issue of antigen escape following CD19 CAR-T cell therapy.

**CD72** is a B cell-specific protein that recruits Src homology region 2 domain-containing phosphatase-1 (SHP-1) to intracellular ITIM motifs and is involved in the negative regulation of BCR signaling [195,196]. CD72 was shown to interact with CD5, CD100, and nuclear lupus self-antigens [197,198,199]. The interaction with CD100 turns off the negative signals of CD72 and enhances BCR signaling [198]. CD72 −/− mice develop lupus-like syndrome and CD72 is downregulated in humans diagnosed with lupus erythematosus, indicating the involvement of CD72 in the pathogenesis of autoimmune diseases [200,201]. CD72 is predominantly expressed in B cells. Its expression starts at the pro-B cell stage and continues throughout all stages of B cell development with the exception of plasma cells [146]. CD72 is also abundantly expressed in B cell-derived neoplasms, including B-ALL, CLL, and B cell lymphomas [146,147,202]. In contrast, CD72 is rarely expressed in other normal tissues including the human brain [146,147]; however, the presence of CD72 was reported on a subset of activated NK cells [203] and in mast cells [204]. Recently, Nix et al. analyzed the surfaceome of a MLL-rearranged B-ALL subtype, in which significant cell surface upregulation of CD72 was observed [147]. Using an in vitro yeast display library, the authors developed CD72-binding nanobodies and generated CD72 CAR-T cells effective against CD72-positive B-ALL and B cell lymphoma cell lines. Importantly, CD72 expression was maintained in CD19-negative cells and CD72 CAR-T cells effectively eliminated CD19-negative leukemia cells in vitro and in vivo. In contrast, CD72 CAR-T cells were not toxic against normal cells including peripheral blood mononuclear cells, immortalized vascular endothelial cells, mesenchymal stem cells, and induced pluripotent stem cells [147]. Overall, CD72 CAR-T cells are very promising candidates to be tested in clinical trials, in particular as a second-line treatment in patients relapsing after CD19-targeted immunotherapy.

### 5.2. Promising Antigens Tested in Clinical Trials

**B cell activating factor receptor** (BAFF-R, also known as BR3, CD268 or TNFRSF17) is a type III membrane protein belonging to the tumor necrosis factor receptors (TNFR) family [205]. The BAFF-R interaction with its specific ligand BAFF was shown to be crucial for the generation of mature B cells in both mice and humans [206,207,208]. In humans, the receptor can be detected on most B cells. The evaluation of BAFF-R expression on different B cell subsets revealed that cells with pre–B phenotype as well as plasma cells lack the receptor, while most circulating B cells, a subset of B cells within germinal centers, and B cells colonizing the mantle zones of reactive lymphoid tissues and a splenic marginal zone are BAFF-R-positive. Importantly, the BAFF-R expression pattern was found to be preserved on mature neoplastic B cells [159]. BAFF-R protein is present on several lymphomas, including MCL, FL and DLBCL [209,210,211,212,213], and on most CLL cells [214]. Surprisingly, malignant pre-B cells also exhibit high BAFF-R expression in contrast to their normal counterparts [215,216]. Moreover, BAFF-R-positive blasts were found to persist in previously treated relapsed pediatric B-ALL [217]. High tissue specificity and the low possibility of BAFF-R antigen escape related to its importance for B cell survival make this receptor an attractive target for immunotherapies in both lymphomas and leukemias.

Antibodies targeting BAFF-R were demonstrated to promote NK cell-mediated killing of B-ALL, CLL and MCL cells in pre-clinical studies [213,218,219], and currently, anti-BAFF-R antibody ianalumab (VAY736) is being evaluated in the treatment of CLL patients in combination with ibrutinib (NCT03400176). In 2019, the results with BAFF-R-specific CAR-T cells were firstly published [220]. In this study, BAFF-R-targeting CAR-T cells caused tumor regression and prolonged the survival of mice with various human lymphomas and B-ALL. Moreover, BAFF-R CAR-T cells efficiently eradicated CD19-negative malignant cells, which were either generated using CRISPR-Cas9 technology, or obtained from the patients relapsing after CD19-targeted therapy. In 2020, a first-in-human trial evaluating BAFF-R-targeting CAR-T cells for patients with r/r B-ALL was initiated (NCT04690595).

**CD37** belongs to the transmembrane 4 superfamily (TM4SF) of tetraspanin proteins, which have four potential membrane-spanning domains. Unlike most tetraspanin proteins, which are not tissue-specific, CD37 can be detected only in several subsets of immune cells [221] and plays a role in both cellular and humoral immune responses [222,223]. B cells show the highest expression of CD37 among leukocytes in human blood and in primary and secondary lymphoid organs [166]. Low or medium CD37 expression was also observed on T cells, NK cells, monocytes and dendritic cells. Importantly, the level of CD37 on B cells changes during their development, with low expression in B cell precursors, a peak in mature/peripheral B lymphocytes and a decrease at the stage of plasma cells [224]. A similar pattern of expression is observed on malignant B cells, as B-ALL and MM cells were found to be CD37-low or CD37-negative, whereas mature/peripheral B cell leukemias and lymphomas highly express this antigen [224]. Therefore, CD37 is considered to be a promising immunotherapeutic target in non-Hodgkin lymphoma (NHL) and CLL [225]. Several anti-CD37 molecules were investigated in clinical trials, including monoclonal antibody otlertuzumab [226] (NCT00614042, NCT01188681), Fc-engineered monoclonal antibody BI 836826 [227] (NCT01403948), antibody-drug conjugates AGS67E [228] (NCT02175433), IMGN529 [229] (NCT01534715), and radio-immunoconjugate betalutin [230] (NCT02658968). The first CD37-specific CAR was presented in 2018 and its efficacy in the eradication of MCL tumors was demonstrated in murine models, which were established using a Jeko-1 cell line and a patient-derived xenograft [231]. Importantly, although CD37 may also have been present on T cells, no significant T cell fratricide occurred during the in vitro expansion of CD37-CAR T cells. These results were also confirmed by Köksal et al., along with the additional observation that CD19 and CD37 are not always mutually expressed on the cell surface, and that CD37 CAR-T cells are able to kill both CD19-positive and CD19-negative cells [232]. Since the pre-clinical studies showed no evidence of the off-tumor activity, the approach was advanced to a clinical trial, where CD37 CAR-T cells were evaluated in patients with relapsed or refractory CD37-positive hematologic malignancies (NCT04136275). According to the recent report, three out of four enrolled patients with relapsed B cell or T cell malignancies eventually achieved CR; however, one patient with refractory double-hit high-grade B cell lymphoma (HGBCL), who relapsed after CD19 CAR-T, experienced disease progression post CD37 CAR-T administration with a CD19-negative and CD37-negative phenotype. Additionally, two patients experienced unexpected bone marrow aplasia, but it was rescued with allogeneic HSCT [233].

**GPRC5D** is a seven-pass membrane protein belonging to the family of orphan G-protein coupled receptors, all of which lack identified ligands [234]. In normal tissues, elevated levels of *GPRC5D* mRNA were observed in differentiating cells that produce hard keratin [235], in lymph nodes, and the spleen. Minimal *GPRC5D* mRNA levels were also detected in lung, skin, and testis [236]. However, GPRC5D protein was found on the surface of the cells from hair follicles [173], and among hematopoietic compartment only on CD19-positive B cells and plasma cells [174,175]. In 2012, *GPRC5D* mRNA was for the first time reported to be expressed in the bone marrow of patients with MM [237]. Moreover, it was later demonstrated that MM cells express significantly higher levels of GPRC5D mRNA and protein as compared to normal plasma cells, and, importantly, MM patients’ plasma cells continue to express GPRC5D after therapy with immunomodulatory drugs, proteasome inhibitors, or CD38-targeting antibodies [175]. The GPRC5D surface protein level was also confirmed in several MM cancer cell lines [236]. Pre-clinical studies of talquetamab (JNJ-64407564), a humanized bispecific antibody binding CD3 and GPRC5D, demonstrated its ability to effectively induce MM cell death and inhibit tumor growth in murine models [236]. Moreover, an ex vivo analysis showed that MM cells isolated from newly diagnosed or heavily pretreated refractory patients were equally susceptible to talquetamab-mediated lysis [175]. The initial results of an ongoing clinical study of talquetamab (NCT03399799) enrolling 137 patients presented an overall response rate (ORR) at 78% in patients who received the drug intravenously [238]. GPRC5D was also recognized as an attractive molecule for CAR-based therapy, and in 2018, GPRC5D-specific CAR was shown to effectively target MM cells in vitro and eradicate MM xenografts in a murine model [239]. Moreover, the ability of GPRC5D CAR-T cells to eliminate BCMA KO MM cells in vivo was observed by Smith et al. along with an independent BCMA and GPRC5D protein expression in MM patients’ CD138-positive plasma cells [173]. Altogether, these results suggest that treatment with anti-GPRC5D CAR-T cells may rescue relapses caused by an antigen escape after BCMA CAR-T therapy. The GPRC5D-targeted CAR construct MCARH109 is currently being evaluated in a clinical trial (NCT04555551), and the first results were shown in 2021. So far, 18 r/r MM patients were enrolled in the study. From 12 patients who had already received the treatment, 83% of them had at least a minimal response and 75% remained progression-free (13 weeks median follow-up). No dose-limiting toxicities were observed during the treatment. Additionally, six patients who experienced relapse following BCMA CAR-T therapy responded to MCARH109, and two of them achieved stringent CR [240].

### 5.3. New Directions for Alternative CAR-T Targets Development

In the light of lessons learned from the experience with single-aimed CAR-T regimens, the field of adoptive therapies is now focused on developing multiple-targeting CAR-T cells. Currently, the safety and efficacy of the approach is being evaluated in a clinical study (NCT03455972), where patients with MM receive both CD19 and BCMA CAR-T cells [241]. The more advanced approach involves utilizing CAR constructs able to recognize multiple targets simultaneously. The treatment with dual CAR-T targeting both CD19 and BCMA resulted in CR in 9/16 MM patients (56.3%) with the median follow-up time being 7.3 months (NCT04236011) [242]. In addition, CD38 and BCMA bispecific CAR-T cells application led to stringent CR in 13 out of 16 patients, 76.9% of whom did not relapse or progress during a follow-up of 11.5 months (ChiCTR1900026286) [243]. In non-Hodgkin lymphoma or CLL, bispecific CARs targeting simultaneously CD19 and CD20 were also successful, with 92% of the patients having CR (NCT03019055). Importantly, the relapse in some of the patients was not associated with CD19 antigen loss [244]. In the case of B-ALL, CD19 and CD22 dual CAR-T cells were evaluated in clinical trials (NCT03233854, NCT03289455). Despite the promising results from in vitro and in vivo studies [245], relapses were observed in approximately 50% of the patients [246,247].

Moreover, it was recently reported that CD20 and CD22 are not only heterogeneously expressed in B-ALL samples but can also be downregulated in CD19-negative relapsed cancer cells [98]. The solution to prevent cancer cell escape via such a mechanism could be the induction of the antigen expression pharmacologically. For instance, bryostatin 1 potently upregulated CD22 expression and improved CD22 CAR-T activity in a pre-clinical B-ALL model [248]. In addition, histone deacetylase inhibitors are known to upregulate CD20 expression on several types of malignant B cells [249,250], though not on CLL cells in vivo [251].

The limitation for a traditional CAR construct in terms of specificity is its ability to recognize molecules expressed only on the cell surface; however, recent advances in the construct design may overcome this limitation by generating TCR-like CARs. TCRs can detect particular tumor antigens as peptides presented by MHC molecules. Since most of the mutated proteins are presented in this mechanism, TCR-based CARs can be more tumor-specific and thus less toxic to non-malignant cells [252]. The examples are novel TCR-like CAR-T cells targeting an HLA*0201-restricted SSX2 epitope, which were shown to be effective against acute myeloid leukemia cells in vitro [253]. In addition, TÜ165 CAR-T is able to recognize an Epstein–Barr nuclear antigen (EBNA)−3C-derived peptide in HLA-B*35 context and specifically target EBV-infected B cells [254].

## 6. Conclusions

Adoptive CAR-T cell therapy has emerged as a breakthrough treatment for relapsed and refractory B cell malignancies. Despite initial success and an induction of remissions, long-term observations have provided data showing a significant number of patients with disease progression and relapse. Hence, understanding the major difficulties at all phases of CAR-T treatment is of crucial importance.

Clinical trials evaluating CD19 CAR-T and their follow-ups have already shed more light on the treatment-related obstacles and the approaches that could be undertaken to avoid them. Therefore, the current research focuses on improving various aspects, including CAR-T cells proliferation and persistence, their killing potential in an immunosuppressive tumor environment, and target antigen expression. Importantly, relapses associated with the loss of target antigen following CAR-T cells administration have led to an interest in finding alternative targets. One of the most important characteristics of a potent target is its tumor-specificity. Unfortunately, such targets are not broadly available as many of them are also expressed on normal hematopoietic cells or other tissues. Hence, many studies develop strategies to address off-tumor effects, for instance by modulating scFv affinity, incorporating on/off switches or suicide genes to the CAR constructs, or the generation of TCR-based CARs. In addition, high-throughput approaches are currently being implemented to study the surfaceome of malignant cells. Such a large-scale approach generating extensive data can yield positive results and help to identify yet unexplored surface antigens for CAR-T cell therapy. In this review, we described several antigens that were selected either by standard or large-scale approaches and that are currently under investigation in pre-clinical or clinical trials. However, targeting only one antigen on cancer cells poses a risk, as the administration of single-targeting CD19 CAR-T cells was linked with unsatisfactory relapse rates. For this reason, the selection of an antigen that is involved in malignant cells’ survival, and that its loss is detrimental, would be of great advantage. Another alternative to solving the issue of target antigen downmodulation is to target multiple antigens with a single CAR-T cell, and such attempts are already being implemented in clinical trials.

Even though CAR-T cell therapy still requires improvement, it has already revolutionized the care of patients with blood cancers; however, further basic research, as well as clinical trials, are needed to find optimal therapeutic solutions and durable effects. Ideally, all those efforts would lead to a safe, effective, and financially available adoptive therapy for all patients with hematological malignancies.

## Figures and Tables

**Table 1:** Clinically approved CAR-T therapies in B cell and plasma cell-derived malignancies.

Generic Name and Brand Name	Indication	Target (Single Chain Variable Fragment, scFv)	Signaling Domain/ Hinge and TM	Gene Editing Vector	First FDA/EMA Registration Date	Approval-Based Clinical Trials, Number of Participants	CR or ORR Rate	Long-Term Follow-Up Studies, Number of Participants	Survival (Median Follow-Up in Years)
Tisagenlecleucel (KYMRIAH)	R/R pediatric and YA B-ALL	CD19 (FMC63)	4-1BB-CD3ζ/CD8α	Lentivirus	August 2017/ August 2018	ELIANA, *n* = 75 [NCT02435849] [12]	CR 60%	NCT01593696, *n* = 50 [13]	median OS 10.5 months (4.8 years)
	R/R adult DLBCL				May 2018/ August 2018	JULIET, *n* = 93 [NCT02445248] [14]	CR 40%	JULIET follow-up, n = 115 [15]	median OS 11.1 months (3.4 years)
Axicabtagene Ciloleucel (YESCARTA)	R/R adult DLBCL (including DLBCL arising from FL)	CD19 (FMC63)	CD28-CD3ζ/CD28	Retrovirus	October 2017/ August 2018	ZUMA-1, *n* = 101 [NCT02348216] [16]	CR 54%	ZUMA-1 follow up, *n* = 101 [17]	median OS 25.8 months (4 years)
	R/R adult PMBCL								
	R/R adult FL				Mar 2021/ not yet registered	ZUMA-5, *n* = 84 [NCT03105336] [18]	CR 79%	not yet available	not yet available
Brexucabtagene Autoleucel (TECARTUS)	R/R adult MCL	CD19 (FMC63)	CD28-CD3ζ/CD28	Retrovirus	July 2020/ December 2020	ZUMA-2, *n* = 60 (efficacy group) [NCT02601313] [19]	CR 67%	not yet available	not yet available
	R/R adult B-ALL				October 2021/ not yet registered	ZUMA-3, *n* = 55 [NCT02614066] [20]	CR 56%	not yet available	not yet available
Lisocabtagene Maraleucel (BREYANZI)	R/R adult DLBCL (including DLBCL arising from indolent lymphoma)	CD19 (FMC63)	4-1BB-CD3ζ/IgG4 and CD28	Lentivirus	May 2021/ pending registration	TRANCEND NHL 001, n = 256 [NCT02631044] [21]	CR 53%	TRANCEND NHL 001 follow-up, *n* = 257 [22]	median OS 27.3 months (2.4 year)
	R/R adult PMBCL								
	R/R FL3B								
Idecabtagene Vicleucel (ABECMA)	R/R MM	BCMA	4-1BB-CD3ζ/CD8α	Lentivirus	Mar 2021/ August 2021	KarMMa, *n* = 100 (efficacy group) [NCT03361748] [23,24]	ORR 72% sCR 28%	not yet available	not yet available

Note: Above 2 years of follow-up was considered long-term. Abbreviations: B-ALL—B cell acute lymphoblastic leukemia, CR—complete response, DLBCL—diffuse large B cell lymphoma, FL—follicular lymphoma, FL3B—follicular lymphoma grade 3B, MCL—mantle cell lymphoma, MM—multiple myeloma, ORR—overall response rate, OS—overall survival, PMBCL—primary mediastinal large B cell lymphoma, R/R—relapse/refractory, sCR—stringent complete response, YA—young adults.

**Table 2:** Alternative targets for CAR-T therapy in B cell and plasma cell-derived malignancies (listed alphabetically).

Targeted Molecule	Physiological Functions	Expression in Non-Malignant Cells [References]	Targeted Disease	References or Numbers of Clinical Trials
***pre-clinical studies***				
CD23	regulation of IgE responses	a subset of T and B cells, monocytes, leukocytes, follicular DCs, intestinal epithelial cells, bone marrow stromal cells [129,130,131,132,133,134,135]	CLL	[136,137]
CD32B	regulation of B cell activation, antibody production	B cells, DCs, granulocytes, liver endothelial cells, airway smooth muscle cells [138,139,140]	CLL	[141]
CD70	T cell activation and proliferation (cytokine, CD27 ligand)	subsets of activated T cells, B cells, NK cells, mature DCs, epithelial cells of the thymic medulla [142,143]	B cell lymphoma	[144,145]
CD72	B cell proliferation, B cell differentiation, negative regulation of BCR signaling	B cells [146]	B-ALL	[147]
CD133	unclear, a marker of undifferentiated cells	hematopoietic stem and progenitor cells [148]	B-ALL	[149,150]
FcμR	IgM receptor	subsets of B cells, T cells, NK cells [151]	CLL	[152]
Siglec-6	inhibition of immune response	memory B cells, exhausted B cells, placenta, mast cells [153,154,155]	CLL	[156]
TSLPR	T and B cell development	DCs, monocytes [157]	B-ALL	[158]
***clinical trials***				
BAFF-R	promotion of B cell survival	B cells (except plasma cells), memory T cells [159]	B-ALL	NCT04690595
CD20	B cell proliferation, B cell differentiation	pre-B cells and mature B cells [160,161]	CLL, B cell lymphoma	NCT04169932
				NCT04030195
				NCT03664635
CD22	regulation of BCR signaling, B cell migration	B cells [162,163]	B-ALL, B cell lymphoma	NCT04546906
				NCT04088864
				NCT04088890
				NCT04007978
				NCT03262298
				NCT04340167
				NCT03244306
				NCT04571138
				NCT02650414
				NCT02315612
				NCT04150497
				NCT03620058
CD30	possibly T cell survival and/or establishment of memory responses	a subset of activated T and B lymphocytes [164,165]	DLBCL, Hodgkin lymphoma	NCT04526834
				NCT03049449
CD37	regulation of humoral and cellular immune responses	mature B cells [166]	CLL, B cell lymphoma	NCT04136275
CD38	cell adhesion and migration, generation of nucleotide metabolites	T cells, B cells, NK cells, plasma cells, monocytes [167]	B-ALL, MM	NCT03754764
				NCT03464916
CD70	T cell activation and proliferation (cytokine, CD27 ligand)	APCs, activated T and B cells [142,143]	MM	NCT04662294
CD79B	promotion of B cell survival	B cells [168]	B-ALL, B cell NHL	NCT04609241
CD123	IL-3 signal transmission, regulation of hematopoiesis	Basophils, plasmacytoid DCs [169,170]	B-ALL	NCT04318678
CD138	cell adhesion, endocytosis	epithelial cells, hepatocytes, plasma cells [171,172]	MM	NCT03672318
GPRC5D	hard keratinization	epithelial cells of the hair follicles, plasma cells, B cells [173,174,175]	MM	NCT05016778
LMP1	induction and maintenance of virus latency by modulation of host’s immune responses	EBV-infected cells [176]	B cell lymphoma	NCT04657965
ROR1	cell differentiation, proliferation and survival	various cells during embryogenesis [177]	CLL, MCL, ALL	NCT02706392
SLAMF7	inhibition of proinflammatory immune responses	B cells, T cells, DCs, NK cells, monocytes [178,179]	MM	NCT04499339
				NCT03958656

Abbreviations: APCs—antigen presenting cells, BAFF-R—B cell activating factor receptor, DCs—dendritic cells, FcμR—immunoglobulin M Fc receptor, GPRC5D—G protein-coupled receptor class C group 5 member D, LMP1—latent membrane protein 1, ROR1—receptor tyrosine kinase like orphan receptor 1, SLAMF7—signaling lymphocytic activation molecule F7, TSLPR—thymic stromal lymphopoietin protein receptor.

## Data Availability

Not applicable.
